# Performance evaluation of metaheuristics-tuned recurrent neural networks for electroencephalography anomaly detection

**DOI:** 10.3389/fphys.2023.1267011

**Published:** 2023-11-14

**Authors:** Dejan Pilcevic, Milica Djuric Jovicic, Milos Antonijevic, Nebojsa Bacanin, Luka Jovanovic, Miodrag Zivkovic, Miroslav Dragovic, Petar Bisevac

**Affiliations:** ^1^ Clinic for Nephrology, Military Medical Academy, University of Defense, Belgrade, Serbia; ^2^ Innovation Center of the School of Electrical Engineering, Belgrade, Serbia; ^3^ Department of Informatics and Computing, Singidunum University, Belgrade, Serbia; ^4^ School of Dental Medicine, University of Belgrade, Belgrade, Serbia

**Keywords:** RNN, EEG anomaly detection, metaheuristics optimization, time series prediction, sine cosine algorithm

## Abstract

Electroencephalography (EEG) serves as a diagnostic technique for measuring brain waves and brain activity. Despite its precision in capturing brain electrical activity, certain factors like environmental influences during the test can affect the objectivity and accuracy of EEG interpretations. Challenges associated with interpretation, even with advanced techniques to minimize artifact influences, can significantly impact the accurate interpretation of EEG findings. To address this issue, artificial intelligence (AI) has been utilized in this study to analyze anomalies in EEG signals for epilepsy detection. Recurrent neural networks (RNNs) are AI techniques specifically designed to handle sequential data, making them well-suited for precise time-series tasks. While AI methods, including RNNs and artificial neural networks (ANNs), hold great promise, their effectiveness heavily relies on the initial values assigned to hyperparameters, which are crucial for their performance for concrete assignment. To tune RNN performance, the selection of hyperparameters is approached as a typical optimization problem, and metaheuristic algorithms are employed to further enhance the process. The modified hybrid sine cosine algorithm has been developed and used to further improve hyperparameter optimization. To facilitate testing, publicly available real-world EEG data is utilized. A dataset is constructed using captured data from healthy and archived data from patients confirmed to be affected by epilepsy, as well as data captured during an active seizure. Two experiments have been conducted using generated dataset. In the first experiment, models were tasked with the detection of anomalous EEG activity. The second experiment required models to segment normal, anomalous activity as well as detect occurrences of seizures from EEG data. Considering the modest sample size (one second of data, 158 data points) used for classification models demonstrated decent outcomes. Obtained outcomes are compared with those generated by other cutting-edge metaheuristics and rigid statistical validation, as well as results’ interpretation is performed.

## 1 Introduction

Electroencephalography (EEG) is a diagnostic method that determines and measures brain waves and brain activity. As a non-invasive, painless and relatively cheap method, it has a wide diagnostic application. The most common indication for EEG is diagnosing or monitoring different types of epilepsy, but it can also be used in diagnosing numerous other neurological disorders such as vascular diseases, tumor processes, infectious diseases, degenerative brain diseases (dementia, Parkinson’s disease, ALS), sleep disorders, narcolepsy, etc. The method has been successfully applied as a scientific tool for almost 100 years (Berger, 1929). There are two primary methods for collecting EEG data. Intracranial EEG Jobst et al. (2020) involves a surgical procedure that places electrodes on to the surface of the brain. However, a more popular method for catapulting EEG signals is the use of non invasive scalp EEG. As the latte in non-invasive it is preferred.

Although EEG is a very detailed and precise method of measuring the electrical activity of the brain, certain factors (such as environmental influences that act during the test itself) can affect a completely objective and realistic picture and interpretation of EEG records. In general, the main problem is represented by artifacts - technical and biological. The main sources of technical artifacts are primarily external audio and visual stimuli from the environment - room temperature, incoming electric and electromagnetic noises from transmission lines, electric lights or other electromagnetic fields. Poor contact and position of the electrodes (the electric field decreases with the square of the distance from the source, and thus the signal strength) leads to high impedance and thus additionally encourages the electromagnetic influence of artifacts. Inadequate material from which the electrodes are made, wrongly adjusted filters, quantization amplification noises during analog-digital conversion can further problematize adequate EEG analysis. The main sources of biological artifacts are uncontrolled muscle movements (e.g., neck, face), blinking or eye movements of the subject. The effect of sweating (physical discomfort during shooting) can also be problematic. Additional complications can occur when collecting data from patients affected by epilepsy, as it can be difficult to discern neurological activity form involuntary musicale spasms cased by seizures. Capturing data during a seizure episode can also prove difficult as the occurrence can be spontaneous and sporadic. Detecting anomalous neurological activity using an EEG is an efficient and non invasive way for epilepsy diagnosis. Finally, inadequate interpretation by the doctor who interprets the recording despite all the technical achievements that minimize the influence of occurring artifacts can be crucial in the misinterpretation of EEG findings (Müller-Putz, 2020).

As a non-invasive method, EEG can have advantages over other imaging methods, especially when patients have absolute or relative contraindications for contrast (NMR or CT) imaging - allergic reactions, advanced chronic renal insufficiency, uncontrolled diabetes mellitus with the risk of lactic acidosis, etc. The fact that it is safe and significantly cheaper method (does not require the use of contrast) additionally recommends it in the early diagnosis of these diseases, which is of inestimable importance for timely therapy in these most serious diseases. Early diagnosis of the disease is a crucial factor that enables a timely treatment, which is of crucial importance for improving the therapeutic outcome, especially in the population of patients with the most severe progressive neurological diseases (Armstrong and Okun, 2020; Symonds et al., 2021; Goutman et al., 2022; Hakeem et al., 2022).

Preceding works have explored the application of AI for medical diagnosis (Jovanovic et al., 2023a). However, few works have explored the potential of time series classification for anomaly detection in neurodiagnostics. The potential of networks capable of accounting for temporal variables such as recurrent neural networks (RNNs) has yet to be fully explored when applied to EEG. As EEG data is sequential, and the RNN has been specially developed to deal with this class of problem there is notable application potential. This work therefore proposes a methodology based on RNNs for anomaly detection in EEG readings. A dataset is composed of a publicly available ^1^ real-world patient dataset (Andrzejak et al., 2001). The testing dataset consists of segments of normal EEG measurements, anomalous EEG measurements of patients suffering from epilepsy, as well as EEG activity during an active seizure.

Two experiments have been conducted. The first experiment involved detecting anomalous activity and was formulated as a binary classification, as two classes exist - normal and anomalous. The second experiment tackled the problem of determining the type of anomalous activity and was formulated as a multi-class classification problem, as the outcome can be classified as normal, anomalous and seizure. Detailed description is provided in Section 4.1.

To improve the performance of the constructed models, several cutting-edge metaheuristic algorithms have been applied to the challenge of optimizing hyperparameters of RNN as well as selecting the optimal network architecture suited to the task. A modified version of the well-known sine cosine algorithm (SCA) (Mirjalili, 2016) algorithm is introduced specifically for this study. Due to the ability of metahersutic algorithms to tackle even NP-hard problems, metaheuristics are a popular choice for tackling the large search space associated with the selection of RNN hyperparamaters. The test outcomes of the simulations carried in this research have been validated through rigorous statistical testing, and the best-performing models are subjected to interpretation using explainable AI techniques to determine the features that contribute to model decisions.

The primary contributions of this work can be summarized as the following.

• a construction of a combined EEG dataset that can be used for the evaluation of seizure and anomaly detection;
• improvements to the classification methodologies available for handling EEG signals using time-series classification based on RNN;
• a proposal for a modified metaheuristic for tuning RNN for classifying RNN signals;
• the interpretation of the best-performing models in order to determine feature importance when considering anomaly detection;
• the explanation of research on this topic and fill the research gap concerning the use of RNN for EEG signal anomaly detection;

The rest of this manuscript has been structured in the following manner. Section 2 yields the background and literature survey on RNNs, metaheuristics optimization, and the general overview of the applications of AI algorithms in medicine. Section 3 presents the basic SCA algorithm, followed by the proposed alterations of the baseline algorithm. The simulation setup that was used for the experiments is given in Section 4, while the simulation outcomes are shown in Section 5, accompanied by the statistical analysis of the results and top-performing model interpretation. Section 6 summarizes the research, gives suggestions for possible future work, and concludes this manuscript.

## 2 Related works and background

Despite advances in imaging, EEG remains the basic test for the diagnosis of epilepsy. Not only can it confirm the diagnosis (it can also clarify the type of epilepsy), but it can have a role in making therapeutic decisions (e.g., whether to stop treatment in patients without seizures) as well as prognostic significance (e.g., evaluating critically ill patients for possible epileptic status or development of encephalopathy) (Trinka and Leitinger, 2022). Apart from mentioned diseases, this method is also widely used in the early diagnosis of dementia (Al-Qazzaz et al., 2014), Mb Alzheimer’s (Stam et al., 2023), brain tumors (Ajinkya et al., 2021), sleep disorders (Kaskie and Ferrarelli, 2019; Steiger and Pawlowski, 2019), as well as the most severe neurodegenerative diseases (Kidokoro et al., 2020). Artificial intelligence methods show immense potential in detection of different medical conditions.

The improvement of new clinical systems, patient information and records, and the treatment of various ailments are all areas where AI technologies, from machine learning to deep learning, play a critical role. The diagnosis of various diseases can also be made most effectively using AI approaches.

This section first introduces the recurrent neural networks and their most important applications in different domains. Afterwards, a brief survey of the metaheuristics optimization is provided. Finally, the overview of general AI applications in medicine is given.

### 2.1 Recurrent neural networks

A recurrent neural network (RNN) (Jain and Medsker, 1999) is a modified version of a traditional neural network designed to handle sequential data. While it maintains many of the components found in neural networks, such as neurons and connections, an RNN has the additional capability of performing a specific operation repeatedly for sequential inputs through recurrent connections. This allows the RNN to store and utilize information from previously processed values in conjunction with future inputs. When provided with an input sequence *I* = *i*_1_, *i*_2_, *i*_3_, …, *i*_*T*_, the network performs the operation described in Eq. 1 at each step *t*.

$$\left[\begin{array}{c} {\hat{o}}_{t}\\ h_{t} \end{array}\right]=\phi_{W}\left(i_{t},h_{t-1}\right)$$ (1)

where ${\hat{o}}_{t}$ represent the output and *h*_*t*_ denote hidden state at time *t*. A neural network *ϕ*_*W*_ is characterized by a weighted network *W*.

Initially developed to enable artificial neural networks (ANN) (Krogh, 2008) to handle sequences of data, recurrent neural networks utilize recurrent connections to incorporate the influence of previous outputs on future predictions. This unique characteristic makes RNN particularly well-suited for accurate time-series forecasting using simpler neural network architectures. However, when dealing with long data sequences, certain limitations persist, where simple RNN architectures struggle to provide accurate results. To address this challenge, the attention mechanism (Olah and Carter, 2016) offers a promising solution.

RNNs have found numerous applications across various domains due to their ability to handle sequential data and capture temporal dependencies. RNNs are extensively used in NLP tasks, such as machine translation, language modeling, sentiment analysis, speech recognition, and text generation (Jelodar et al., 2020; Sorin et al., 2020; Zhou et al., 2020; Nasir et al., 2021). Long Short-Term Memory (LSTM) and Gated Recurrent Unit (GRU) are popular RNN variants commonly used in NLP tasks (Shewalkar et al., 2019; Sherstinsky, 2020; Yang et al., 2020). They are also well-suited for time series forecasting tasks, such as stock price prediction, weather forecasting, and demand prediction in sales or finance domains, as they are capable of capturing patterns and trends in sequential data (Alassafi et al., 2022; Amalou et al., 2022; Bhoj and Bhadoria, 2022; Freeborough and van Zyl, 2022; Hou et al., 2022; Siłka et al., 2022). RNNs are also frequently employed in speech recognition systems, speech synthesis (text-to-speech), and speaker identification. RNNs can process audio data as a sequence of frames, making them suitable for such tasks (Shewalkar et al., 2019; Zhang et al., 2020; Oruh et al., 2022). Finally, RNNs can be applied to video data for tasks like action recognition, video captioning, and video summarization, where temporal information is crucial for understanding the content (Liu et al., 2019; Yuan et al., 2019; Zhao et al., 2019).

RNNs have also been successful in medical domain. Some of the successful applications include medical pre-diagnostics online support (Zhou et al., 2020), cyber-attack and intrusion detection within the medical Internet of Things devices (Saheed and Arowolo, 2021), MRI and CT images processing tasks (Rajeev et al., 2019; Sabbavarapu et al., 2021; Islam et al., 2022), and medical time series (Li and Xu, 2019; Tan et al., 2020), to name the few.

### 2.2 Metaheuristic optimization

While AI methods, like RNNs and ANN show immense potential, their effectiveness, heavily relies on the initial values assigned to a set of parameters known as hyperparameters. Modern methods offer a wide range of control parameters that enable networks to achieve good overall performance while allowing fine-tuning of internal operations to better suit specific problems. However, manually selecting appropriate values for these hyperparameters can be challenging, as modern methods often involve several dozen parameters. Thus, the use of automated methods becomes crucial to facilitate the selection process. Given the broad range of possible parameter values, this task quickly becomes NP-hard, making it seemingly impossible to solve using traditional methods. Consequently, it is imperative to discover and adapt novel approaches to address this challenge.

Metaheuristic algorithms present a feasible solution to this problem. Rather than employing a deterministic approach, they utilize a search strategy. These algorithms do not guarantee finding the optimal solution in a single run but increase the statistical probability of locating the true optimum with each iteration. By adopting this approach, the feasibility of solving NP-hard problems within a reasonable time frame and with manageable computational resources is enhanced. This characteristic makes metaheuristic optimization algorithms a popular choice for hyperparameter tuning. By defining the selection of hyperparameters as a typical maximization problem, optimal values can be determined, thereby improving algorithm performance by further adjusting behaviors to suit the specific task at hand. Researchers have developed numerous metaheuristic algorithms to tackle diverse problem domains, drawing inspiration from various sources.

Stochastic algorithms, known as metaheuristics, are extensively employed in computer science to address NP-hard problems, as deterministic methods are impractical in such cases. These metaheuristic algorithms can be classified into different categories based on the natural phenomena they emulate to guide the search process. Examples include evolution and ant behavior for nature-inspired methods, physical phenomena like storms and gravitational waves, human behavior such as teaching and learning or brainstorming, and mathematical laws like oscillations of trigonometric functions.

Swarm intelligence algorithms are rooted in the collective behavior of large groups consisting of relatively simple units, such as bird flocks or insect swarms. These groups exhibit remarkably synchronized and sophisticated behavioral patterns during essential survival activities such as hunting, scavenging, breeding, and predator avoidance. Swarm intelligence methods, including ant colony optimization (ACO) (Dorigo et al., 2006), particle swarm optimization (PSO) (Wang et al., 2018), artificial bee colony (ABC) (Karaboga and Basturk, 2007), bat algorithm (BA) (Yang and Gandomi, 2012), and firefly algorithm (FA) (Yang and Slowik, 2020), have proven effective in solving a wide range of NP-hard problems in real-life scenarios. In recent years, a particularly efficient family of metaheuristics has emerged that relies on mathematical functions and their properties to facilitate the search process. Prominent examples within this family include the sine-cosine algorithm (Mirjalili, 2016) and the arithmetic optimization algorithm (AOA) (Abualigah et al., 2021).

The diversity of population-based algorithms stems from the no-free-lunch theorem (NFL) (Wolpert and Macready, 1997), which states that there is no universal approach capable of finding the best solution for all optimization challenges. Therefore, the selection of an appropriate metaheuristic method becomes crucial, as a technique that performs well for one problem may not yield the same level of success for another. Hence, the availability of various metaheuristic methods and the need to adapt the algorithm to the specific optimization task at hand.

Metaheuristic algorithms have many uses in a variety of sectors due to their high performance when taking up general optimizations. Some fascinating examples include credit card fraud detection (Jovanovic et al., 2022a; Petrovic et al., 2022), security and intrusion detection (Jovanovic et al., 2023c; Jovanovic et al., 2022b; Bacanin et al., 2022), cloud-edge computing (Bacanin et al., 2019; Bezdan et al., 2020; Zivkovic et al., 2021b), tackling challenging obstacles in emerging industries (Jovanovic et al., 2023a), forecasting COVID-19 cases (Zivkovic et al., 2021c; Zivkovic et al., 2021a), as well as in healthcare (Bezdan et al., 2021; Zivkovic et al., 2022a; Zivkovic et al., 2022c; Jovanovic et al., 2023b; Jovanovic et al., 2022d; Tair et al., 2022). Moreover, metaheuristics have demonstrated remarkable effectiveness in optimizing time series forecasting (Jovanovic et al., 2022c; Stankovic et al., 2022; Bacanin et al., 2023).

### 2.3 Brief overview of AI applications in medicine

To identify diseases that require early diagnosis, such as those related to skin, heart, and Alzheimer’s, researchers have utilized a variety of AI-based techniques, including machine and deep learning models. In order to reach the best level of accuracy, a backpropagation neural network was utilized in a paper by (Dabowsa et al., 2017) to diagnose skin diseases. Authors in (Uysal and Ozturk, 2020) choose to examine T1-weighted magnetic resonance images in order to analyze dementia in Alzheimer’s using Logistic Regression, K-Nearest Neighbors, Support Vector Machines, Decision Tree, Random Forest, and Gaussian Naive Bayes methods. Artificial intelligence can be successfully applied in monitoring and detecting medical conditions like brain tumors (Bacanin et al., 2023; Kushwaha and Maidamwar, 2022), diabetes (Joshi and Borse, 2016) or COVID-19 (Zivkovic et al., 2022b). In Salehi et al. (2023) authors presented a comprehensive review of the usage of Convolutional Neural Networks (CNN) in the context of medical imaging. The diagnosis and treatment of diseases depend heavily on medical imaging, and CNN-based models have shown considerable gains in image processing and classification tasks. It has been successfully used in liver lesion classification (Frid-Adar et al., 2018) based on computed tomography images and tumor identification (Dhiman et al., 2022). Metaheuristics-driven CNN tuning has proven to be successful in COVID-19 diagnostics as well (Pathan et al., 2021).

Machine/deep learning models for epileptic seizure identification utilizing EEG signals have been introduced in a number of research papers. Support vector machines (SVM), k-nearest neighbor (KNN), artificial neural networks (ANN), convolutional neural networks, and recurrent neural networks are a few examples of commonly used algorithms. A hybrid model employing SVM and KNN was suggested in (Dorai and Ponnambalam, 2010) to categorize EEG epochs into seizure and nonseizure types. In the other paper, to identify epileptic seizure, authors employed genetic algorithms, SVM, and particle swarm optimization (Hassan and Subasi, 2016). The SVM algorithm was successfully used in another research (Lahmiri and Shmuel, 2018) to categorize seizures with 100% accuracy. As mentioned before, CNN was initially employed for image categorization. In the study (Antoniades et al., 2016), the authors examine deep learning for automatically generating features from time-domain epileptic intracranial EEG data, specifically utilizing CNN algorithms. A new deep convolutional network-based technique for detecting epileptic seizures is suggested in (Park et al., 2018). The proposed network uses 1D and 2D convolutional layers and is built for multi-channel EEG signals. It takes into account spatio-temporal correlation, a feature in epileptic seizure identification. In the research (Hussein et al., 2019), authors introduced a deep neural network architecture based on RNN to learn the temporal dependencies in EEG data for robust detection of epileptic seizures. The Long Short-Term Memory (LSTM) network is used by the authors in (Hussein et al., 2018) to demonstrate a deep learning-based technique that automatically recognizes the distinctive EEG features of epileptic episodes.

## 3 Materials and methods

This section first describes the baseline variant of the SCA metaheuristics, and highlights the known drawbacks of the algorithm. Afterwards, the suggested improvements are presented and enhanced version of the algorithm is proposed.

### 3.1 Original algorithm - SCA algorithm

The sine cosine algorithm (SCA) method is a distinct optimization metaheuristic that draws its inspiration from the mathematical properties of trigonometric functions (Mirjalili, 2016). By utilizing sine and cosine functions, the SCA method updates the positions of solutions within the population, leading to oscillations that explore the direct range of the optimal solution. These functions ensure that the solutions undergo variation as their output values are confined to the range of −1 to 1. During the initialization stage, a random number of potential solutions are generated within the search region bounds. Stochastic configurable control variables are employed throughout the algorithm’s execution to guide both exploration and exploitation activities.

To update the positions of individual solutions during both exploration and exploitation, the algorithm utilizes both the sine and cosine functions. The positional formulas for the sine and cosine functions are represented by Eq. 2–3 respectively.

$$X_{i}^{t+1}=X_{i}^{t}+r_{1}\cdot \sin\left(r_{2}\right)\cdot \left|r_{3}\cdot P_{i}^{*t}-X_{i}^{t}\right|$$ (2)

$$X_{i}^{t+1}=X_{i}^{t}+r_{1}\cdot \cos\left(r_{2}\right)\cdot \left|r_{3}\cdot P_{i}^{*t}-X_{i}^{t}\right|$$ (3)

in which $X_{i}^{t}$ and *X* ^*t*+1^ *i* represent the position of the current individual in the *ith* dimension at the *t*th and *t* + 1-th iteration cycles respectively, the parameters *r*_1_, *r*_2_, and *r*_3_ represent pseudo-stochastically generated control parameters. Additionally, P*i denotes the position of the destination point (i.e., the final best estimation of the optimal value) in the *ith* dimension.

By interchanging between the equations mentioned above, as depicted in Eq. 4, the control variable *r*4, which is randomly generated within the range of 0–1, is employed to determine whether the sine or cosine function is utilized during the search process. New values of the pseudo-stochastic control parameters are generated for each segment of an individual within the population.

$$X_{i}^{t+1}=\left\{\begin{array}{c} X_{i}^{t+1}=X_{i}^{t}+r_{1}\cdot \sin\left(r_{2}\right)\cdot |r_{3}\cdot P_{i}^{*t}-X_{i}^{t}|,\;r_{4}<0.5\;\\ X_{i}^{t+1}=X_{i}^{t}+r_{1}\cdot \cos\left(r_{2}\right)\cdot |r_{3}\cdot P_{i}^{*t}-X_{i}^{t}|,\;r_{4}\geq 0.5\; \end{array}\right.$$ (4)

The main SCA parameters *r*_1_, *r*_2_, *r*_3_ and *r*_4_ play a crucial role in influencing the behavior of the algorithm under specific circumstances. Parameter *r*1 governs the movement of the subsequent solution, determining whether it moves away from or towards a designated destination. To enhance the level of randomization and promote exploration, the control parameter *r*2 is set within the range of 0 to 2*π*. The inclusion of parameter *r*3 determines a level of randomness to the movements, emphasizing movement when *r*3 > 1 and reducing it when *r*3 < 1. Additionally, the parameter *r*4 plays a crucial role in determining the selection between the sine or cosine function for a specific iteration.

To achieve a better stability between exploration and exploitation, adaptive adjustments to the function ranges are made according to Eq. 5.

$$r_{1}=a-t\frac{a}{T}$$ (5)

where variable *t* denoting the current iteration, *T* representing the maximum number of iterations per run, and *a* representing a fixed number.

### 3.2 Modified SCA algorithm

The SCA meta-heuristic demonstrates remarkable performance on bound-constrained and unconstrained benchmarks while maintaining simplicity and a limited set of control parameters (Mirjalili, 2016). However, its performance on standard Congress on Evolutionary Computation (CEC) benchmarks reveals a tendency to converge too rapidly towards the current best solutions, resulting in reduced population diversity. This rapid convergence, coupled with its directed search towards the P*, leads to unfavorable outcomes if the initial results are distant from the optimal solution. As a consequence, the algorithm yields unsatisfactory final results as it converges towards a disadvantageous region within the search space.

To tackle the limitations of the original algorithm, this paper presents a modified version of SCA that incorporates two additional procedures to the baseline SCA metaheuristics. These enhancements have been introduced to address the known shortcomings and further improve the performance of the algorithm.

1. The initial population is formed by employing a chaotic initialization of solutions, and
2. A self-adaptive search procedure that alternates the search process between the elementary SCA search and the firefly algorithm (FA) search procedures.

The initial modification suggested for the basic version of SCA involves a chaotic initialization of the initial population. This technique is intended to generate an initial collection of individuals close to the optimal region within the search space. The idea of incorporating chaotic maps into metaheuristic algorithms to enhance the search phase was proposed by (Caponetto et al., 2003). Several other notable studies, such as (Kose, 2018; Wang and Chen, 2020; Liu et al., 2021), have demonstrated that using chaotic sequences for the search procedure yields higher efficiency compared to traditional pseudo-random generators.

Among the various chaotic maps available, empirical simulations conducted with SCA metaheuristics have indicated that the logistic map produces the most promising outcomes. As a result, the modified SCA employs the chaotic sequence *β*, initialized with the pseudo-random value *β*_0_, generated using the logistic mapping according to Eq. 6, at the beginning of its execution.

$$\beta_{i+1}=\mu \beta_{i}\times \left(1-\beta_{i}\right),\;i=0,1,2,\ldots ,N-1,$$ (6)

where *N* and *μ* denote the count of individuals in the population and chaotic control parameter, respectively. The parameter *μ* is initialized to value 4, while respecting the provided set of limits of *β*_0_: 0 < *β*_0_ < 1 and *β*_0_ ≠ 0.25, 0.5, 0.75, 1.

The individual *i* is a subject of mapping with respect to the generated chaotic sequences applied to every component *i* as defined by the following equation:

$$X_{i}^{c}=\beta_{i}X_{i},$$ (7)

where $X_{i}^{c}$ corresponds to the new position of solution *i* after chaotic perturbations.

The complete process of generating the initial population using chaos-based initialization is presented in Algorithm 1. It is essential to highlight that this introduced initialization mechanism does not impact the algorithm’s complexity concerning fitness function evaluations (*FFEs*), as it generates only *N*/2 random solutions initially and then executes mapping of those individuals to the corresponding chaotic-based solutions.

Algorithm 1 Pseudo-code that describes the chaotic-based initialization mechanism.

Step 1: Generate population *Pop* of *N*/2 solutions by employing the conventional initialization method: *X*_*i*_ = *LB* + (*UB* − *LB*) ⋅ *rand* (0, 1), *i* = 1, …*N*, where *rand* (0, 1) represents the pseudo-random number within [0,1] and *LB* and *UB* represent vectors with lower and upper bounds of each solution’s component *i*, respectively.
Step 2: Produce the chaotic population *Pop* ^*c*^ of *N*/2 individuals by mapping the solutions that belong to *Pop* to chaotic sequences by employing Eq. 6–7.
Step 3: Merge *Pop* and *Pop* ^*c*^(*Pop* ∪ *Pop* ^*c*^) and sort merged collection of *N* individuals according to fitness value in ascending order.
Step 4: Establish the current best solution *P*.

The second modification to the baseline SCA is the self-adaptive search method, which governs the switching between the basic SCA search procedure and the FA’s search procedure (Yang, 2009), as represented by Eq. 8.

$$X_{i}^{t+1}=X_{i}^{t}+\beta_{0}\cdot e^{-\gamma r_{i,j}^{2}}\left(X_{j}^{t}-X_{i}^{t}\right)+\alpha^{t}\left(\kappa -0.5\right),$$ (8)

where *α* is a randomization value, and *κ* denotes the arbitrary number taken from the Gaussian distribution. Distance between a pair of fireflies *i* and *j* is denoted by *r*_*i*,*j*_. Additional improvement of the FA’s search capability is achieved by applying the dynamic *α*, as described by (Yang and Slowik, 2020).

The modified SCA method alternates between the SCA and FA search mechanisms for each component *j* belonging to every solution *i* in the following manner: If the generated pseudo-random number within the limits [0,1] is less than the search mode (*sm*), then the *j*th component of solution *i* will update by using the FA search (Eq. 8). Otherwise, the plain SCA search will be employed (Eq. 4). The search mode *sm* control variable determines the balancing among the SCA and FA search procedures, with a higher emphasis on the FA search to update solutions in the early rounds. In the later phases when the search space is explored more extensively, the SCA search will be triggered more frequently. This behavior is enabled by dynamic reduction of the value *sm* during each round *t* as follows:

$$sm_{t}=sm_{t-1}-\frac{t}{T}.$$ (9)

The initial *sm* value was determined empirically, and assigned to 0.8 throughout the experiments in this research.

Modified SCA algorithm in fact represents a low-level hybrid, as it integrates FA search procedure to the SCA algorithm. Novel method was given the name hybrid adaptive SCA (HASCA). The pseudo-code presenting the internal implementation of the suggested algorithm is provided by Algorithm 2.

Algorithm 2 The HASCA pseudo-code.

Generate starting populace of *N* solutions by utilizing chaotic initialization 1.
Tune the control parameters and initialize dynamic parameters
**while** exit criteria has not been met **do**
   **for** each solution *i* **do**
      **for** each component *j* belonging to solution *i* **do**
          Generate arbitrary value *rnd*
      **if** *rnd* < *sm* **hen**
          Update component *j* by executing FA search (Eq. 8)
    **else**
        Update component *j* by executing SCA search (Eq. 4)
       **end if**
    **end for**
   **end for**
   Evaluate the population based on the fitness function value
   Determine the current best solution *P*
   Refresh values of the dynamic parameters
**end while**
Return the best-discovered solution

In conclusion, it is important to highlight that the HASCA does not introduce any additional overhead to the baseline SCA method. The complexity in terms of fitness function evaluations (FFEs) for both the basic and enhanced methods is *O*(*N*) = *N* ⋅ *N* ⋅ *T*, where *N* is the population size and *T* is the number of iterations.

### 3.3 Applied method

The introduced algorithm and the introduced modifications aimed at improving performance are incorporated into a testing framework and compared to several state-of-the-art algorithms as well as the original base algorithms to determine the improvements made. The algorithms are provided with search space constraints for RNN hyperparameters and allocated a population and a certain number of iterations to improve performance. Specific values are presented in the experimental section.

The framework is provided with a dataset of real-world EEG data, further described in the experiential section. A segment of the data is used for training and another for testing. Once hyperparameter optimization is performed, algorithms are evaluated based on objective function as well as other classification metrics described in the experiential setup.

**FIGURE 1:**
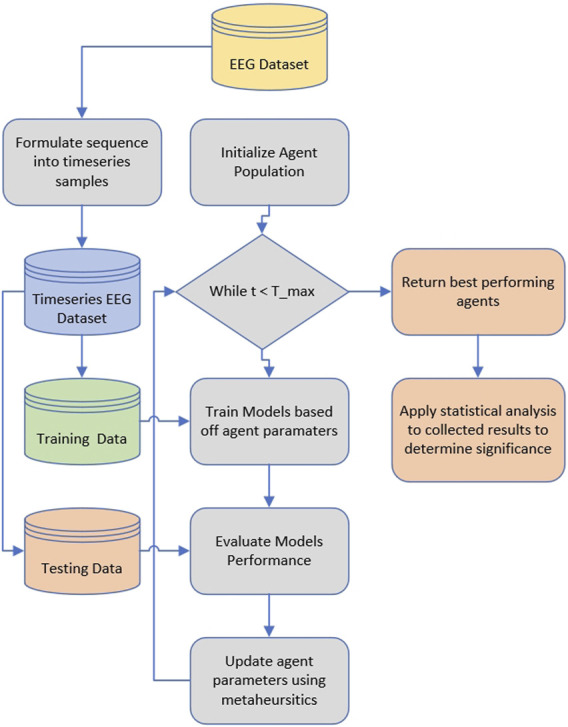
Flowchart of the applied methodology.

Finally, the attained results for all the simulations are subjected to rigorous statistical analysis to determine the statistical significance of the introduced improvements. A flowchart of the described process is presented in Figure 1.

## 4 Experimental setup

This section described the experimental setup of this work. The utilized dataset and preparation procedure are described. This is then followed by the metrics used for the evaluation of each approach. Finally, the experimental setup is provided in details. All simulations have been carried out using the Python programming language and appropriate supporting libraries, TensorFlow, Pandas, Seaborn, and Python SHAP libraries. Experimentation has been carried out on a machine srunning Windows 10. With an Intel i7 CPU, 32 Gb of available RAM memory and a Nvidia 3060 GPU.

### 4.1 Dataset description and prepossessing

When tackling medical data several challenges arise. One major issue is the availability of the dataset. Oftentimes, quality data is not publicly available, limiting research capacity for outside researchers to build upon established techniques. This work therefore uses a publicly available ^2^ labeled dataset Andrzejak et al. (2001). However, while this dataset is properly labeled and well formatted, some reprocessing is needed to make it suited for this research. The original dataset contains EEG data concerning five patients. Two of these are known to be neurotypical individuals labeled A and B in the original data. Two patients, confirmed to be suffering from epilepsy, are labeled C and D in the original data. Finally, the fifth EEG data segment of the dataset was captured during an active epileptic seizure labeled E in the original data. Each sample consists of 26 s of recorded data, totaling 4,096 data points from 100 electrodes.

These data segments have been recombined into two separate subsets used for the two experiments conducted in this research. The first dataset combined neurotypical individuals’ EEG (sample A) readings with that of a person suffering from epilepsy (sample C). Each of the samples was segmented into half-second intervals (158 data points) and randomly recombined to formulate a single continuous EEG reading.

A similar procedure was repeated for the dataset used in the second dataset. However, to better explore the potential of RNNs this experiment was formulated as a multi-class classification. A combination of the available data was created using data from a neurotypical individual (sample A) a patient confirmed to be suffering from epilepsy (patient D) as well as data captured during an active seizure (sample E). The data was once again recombined using half-second intervals (158 data points) and recombined into a single continuous EEG reading.

**FIGURE 2:**
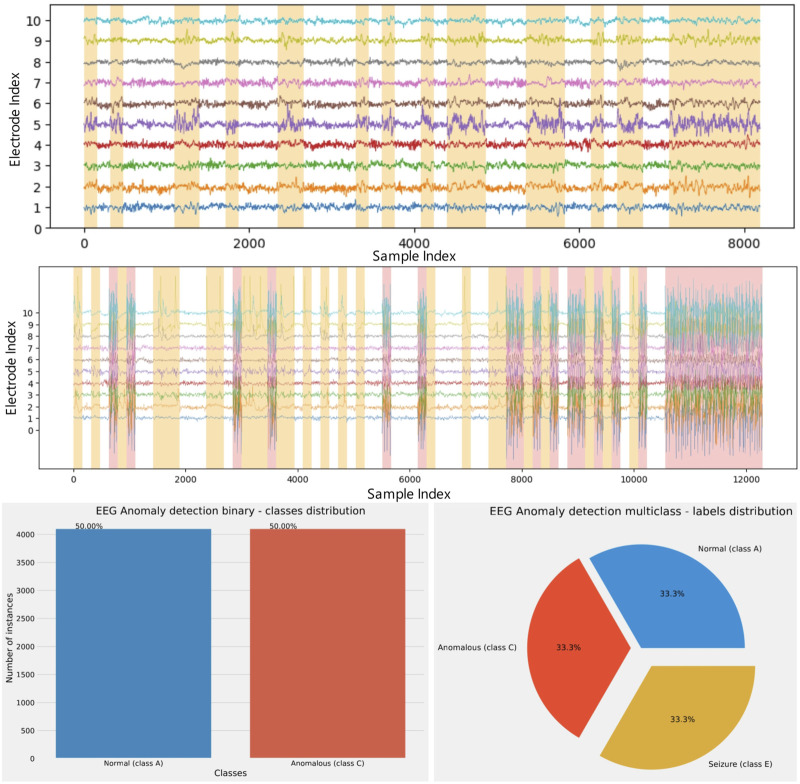
Visualizations of the first ten electrode readings of the constructed binary (top), multi-class (middle) dataset and dataset distribution (bottom) in each experiment.

The readings form the first ten electrodes in the constructed datasets can be seen in Figure 2. In this figure a white background indicates normal readings, an orange background signifies anomalous activity, while a red background indicates readings taken during and active seizure.

In terms of salmon tracking experiments, the first experiment with a dataset representing a combination of classes A (normal) and C (anomalous), using a binary classification approach, will be labeled as ‘experiment 1.’ Meanwhile, the second experiment, which used a dataset representing a combination of classes A (normal), D (anomalous), and E (seizure) and employed a multiclass classification approach, will be referred to as ‘experiment 2’.

### 4.2 Classification metrics

For each tested model, the traditional classification metrics - precision, recall, accuracy and F1-score were evaluated. The following formulas apply to those metrics:

$$Precision=\frac{TP}{TP+FP}$$ (10)

$$Recall=\frac{TP}{TP+FN}$$ (11)

$$Accuracy=\frac{TP+TN}{TP+TN+FP+FN}$$ (12)

$$F_{1}Score=2\times \frac{Precision\times Recall}{Precision+Recall}$$ (13)

where TP and TN stand for true positive and negative, respectively, whereas FP and FN stand for false positive and negative. The error rate was represented by the indicator function 1 − *accuracy*.

The Cohen’s Kappa coefficient was also reported in the experiments (Warrens, 2015). This coefficient, as described in (McHugh, 2012), assesses the inter-rater reliability and can also serve as an evaluation measurement for the performance of the regarded classification models. In contrast to the overall accuracy of the model, which may be deceptive when dealing with imbalanced datasets, Cohen’s Kappa considers the class distribution imbalance to yield more dependable outcomes. This coefficient is calculated according to Eq. 14:

$$\kappa =\frac{p_{o}-p_{e}}{1-p_{e}}=1-\frac{1-p_{o}}{1-p_{e}}$$ (14)

where *p*_*o*_ denotes the observed values, and *p*_*e*_ marks the expected values.

### 4.3 Optimization setup

To facilitate the proper application of ML algorithms, proper hyperparameter values should be selected for the algorithm to yield acceptable performance for the problem being tackled. However, the process of selection can be considered an NP-hard challenge given the large number of possible combinations, rendering traditional methods an inadequate approach. This work utilizes mechanistic optimization to select hyperparameters that attained the desired performance outcomes.

As stated above, two sets of experiments were conducted. The first experiment (experiment 1) focused on detecting anomalous activity, presented as a binary classification problem. The second experiment (experiment 2) dealt with determining the type of anomalous activity, structured as a multi-class classification problem.

For both conducted experiments, the datasets were split in a standard way, 70% was allocated to training, 10% for validation, and finally, the remaining 20% was used to test the approaches. Dataset was normalized. The number of lags was set to 15.

Two types of parameters are optimized in this work. Firstly training parameters of the RNN are selected including the learning rate from a range of [0.0001,0.01] and dropout within the range [0.05,0.2] for both experiments. The number of training epochs was selected from the range of [30,60] for the first experiment, and from the range of [50,150] for the second experiment. It is important to note that an early stopping criterion is also used during training equaling 1/3 of the maximum number of training epochs. Secondly, due to the significant influence of network architecture on performance, RNN structures are optimized. The number of network layers is selected from the range of [1,2] for both experiments. Finally, the number of neurons in each layer was selected from the interval [*lags*/3, *lags*] for the first experiment, and from the interval [*lags*/2, *lags* ⋅ 2] for the second experiment. The values used for the second experiment (training epochs and number of neurons) have been increased due to the increased complexity of the multiclass-classification problem. Parameter ranges have been empirically determined through trial and error and based on previous experience with hyperparameter optimization.

To evaluate the optimization potential of the introduced algorithm, several state-of-the-art metaheuristics have also been tasked with optimizing RNN parameters under identical conditions. The tested algorithms include the original SCA (Mirjalili, 2016) as well as the GA (Mirjalili and Mirjalili, 2019), PSO (Kennedy and Eberhart, 1995), FA (Yang, 2009), BSO (Shi, 2011), RSA (Abualigah et al., 2022), and COLSHADE (Gurrola-Ramos et al., 2020). Each metaheuristic was issued a total of six individual agents and allowed eight iterations to improve performance. Finally, to facilitate statistical analysis and account for randomness associated with metaheuristic algorithms, testing was carried out through 30 independent runs.

The classification error was used as the objective function for both experiments, since datasets are balanced. Additionally, values of the Cohen Kappa indicator are presented as well.

## 5 Experimental outcomes, comparative analysis, validation and interpretation

This section brings forward the detailed simulation outcomes for both executed experiments. Afterwards, the statistical analysis of the experimental outcomes has been conducted to determine if the performance improvements are statistically significant. Finally, the best model’s interpretation is provided at the end of this section. In all tables that contain simulation results, the best score in every category is marked bold.

### 5.1 Experiment 1 - Binary classification

The simulation outcomes of the proposed RNN-HASCA method and seven competitor methods for the binary classification problem are summarized in Table 1. Again, it is worth highlighting that the objective function was classification error, and the scores of the Cohen’s Kappa coefficient are provided as well. When observing the overall metrics of the objective function across 30 independent executions, shown in Table 1, it is possible to note that several metaheuristics algorithms were capable to reach the same best value. However, due to the stochastic nature of metaheuristics algorithms, important metrics are also the worst and mean results, where the proposed HASCA algorithm exhibited superior performance. Regarding the worst metric, SCA, PSO, BSO and RSA finished second, behind HASCA. Additionally, regarding the mean metrics, PSO attained second place, while RSA finished third. It is also worth noting that RNN-HASCA method obtained the best scores for Cohen’s Kappa coefficient as well.

**TABLE 1:** Experiment 1 - overall objective and Cohen Kappa metrics for 30 runs.

Method	Best	Worst	Mean	Median	Std	Var
RNN-HASCA	0.001231	**0.001846**	**0.001436**	0.001231	2.90E-04	8.42E-08
RNN-SCA	0.001846	0.002462	0.002051	0.001846	2.90E-04	8.42E-08
RNN-GA	0.001231	0.003077	0.002051	0.001846	7.68E-04	5.89E-07
RNN-PSO	0.001231	0.002462	0.001641	0.001231	5.80E-04	3.37E-07
RNN-FA	0.001231	0.004308	0.002256	0.001231	1.45E-03	2.10E-06
RNN-BSO	0.001846	0.002462	0.002256	0.002462	2.90E-04	8.42E-08
RNN-RSA	0.001231	0.002462	0.002022	0.002462	5.85E-04	3.43E-07
RNN-COLSHADE	0.001231	0.004308	0.003077	0.003692	1.33E-03	1.77E-06
Cohen Kappa metrics						
RNN-HASCA	0.996072	**0.994114**	**0.995419**	0.996072	**9.23E-04**	**8.51E-07**
RNN-SCA	0.994114	0.992124	0.993451	0.994114	9.38E-04	8.80E-07
RNN-GA	0.996072	0.990143	0.993443	0.994114	2.47E-03	6.08E-06
RNN-PSO	0.996072	0.992143	0.994762	0.996072	1.85E-03	3.43E-06
RNN-FA	0.996072	0.986332	0.992825	0.996072	4.59E-03	2.11E-05
RNN-BSO	0.994100	0.992124	0.992783	0.992124	9.32E-04	8.68E-07
RNN-RSA	0.996072	0.992124	0.992124	0.992124	1.88E-03	3.53E-06
RNN-COLSHADE	0.996072	0.986234	0.990183	0.988243	4.24E-03	1.80E-05

In all tables that contain simulation results, the best score in every category is marked bold.

Detailed metrics achieved in the best individual run of every observed method are provided in Table 2. It must be highlighted that all observed algorithms attained respectable results. Finally, Table 3 depicts the best set of RNN hyperparameters obtained by each algorithm. It is noteworthy that all methods determined the networks with one layer.

**TABLE 2:** Experiment 1 - detailed metrics of the best run of each algorithm.

Metric	RNN-HASCA	RNN-SCA	RNN-GA	RNN-PSO	RNN-FA	RNN-BSO	RNN-RSA	RNN-COLSHADE
Accuracy (%)	99.8769	99.8154	99.8769	99.8769	99.8769	99.8154	99.8769	99.8769
Precision Normal	0.996835	0.993691	0.996835	0.996835	0.996835	0.996825	0.996835	0.996835
Precision Anomaly	0.999236	0.999235	0.999236	0.999236	0.999236	0.998473	0.999236	0.999236
W.Avg. Precision	0.998769	0.998157	0.998769	0.998769	0.998769	0.998153	0.998769	0.998769
Recall Normal	0.996835	0.996835	0.996835	0.996835	0.996835	0.993671	0.996835	0.996835
Recall Anomaly	0.999236	0.998472	0.999236	0.999236	0.999236	0.999236	0.999236	0.999236
W. Avg. Recall	0.998769	0.998154	0.998769	0.998769	0.998769	0.998154	0.998769	0.998769
F1-score Normal	0.996835	0.995261	0.996835	0.996835	0.996835	0.995246	0.996835	0.996835
F1-score Anomaly	0.999236	0.998854	0.999236	0.999236	0.999236	0.998855	0.999236	0.999236
W. Avg. F1-score	0.998769	0.998155	0.998769	0.998769	0.998769	0.998153	0.998769	0.998769

**TABLE 3:** Experiment 1 - best determined RNN parameters.

Method	Learning rate	Dropout	Epochs	Number of layers	Layer 1	Layer 2
RNN-HASCA	0.010000	0.050000	60	1	5	6
RNN-SCA	0.008531	0.050000	60	1	11	10
RNN-GA	0.002725	0.190960	60	1	15	5
RNN-PSO	0.004895	0.066932	60	1	15	15
RNN-FA	0.010000	0.200000	60	1	15	8
RNN-BSO	0.010000	0.200000	43	1	15	10
RNN-RSA	0.008858	0.064926	60	1	14	10
RNN-COLSHADE	0.005873	0.144412	60	1	12	8

**FIGURE 3:**
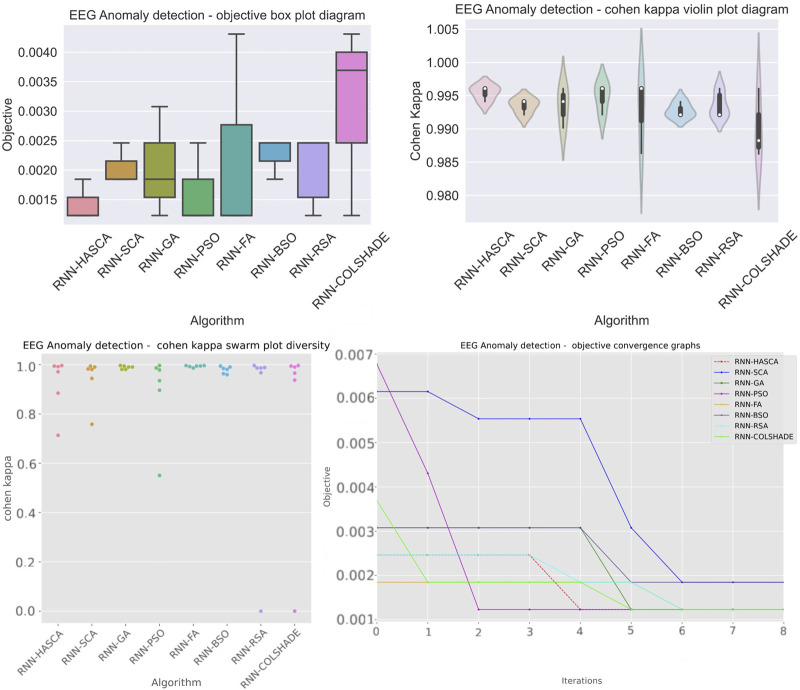
Experiment 1 - box plot, violin plot, swarm diversity diagram and objective convergence diagram.

**FIGURE 4:**
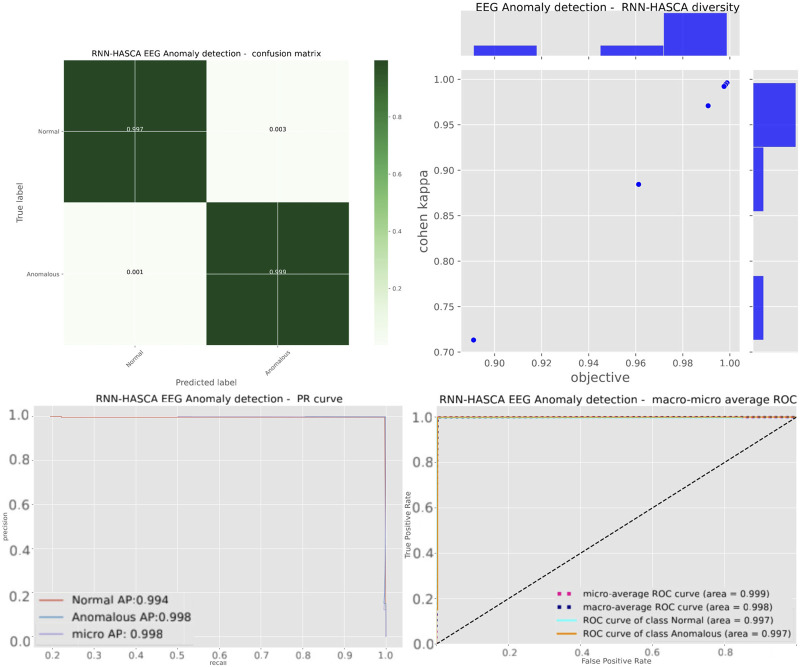
Experiment 1 - confusion matrix, objective indicator joint plot, PR and ROC curves of the proposed HASCA method.

Aiming to present the obtained results clearer, Figure 3 shows the box plots, violin plots, convergence diagram and swarm diversity plots. It can be observed that the HASCA method exhibits satisfactory converging speed, and also the box plots show that the results are very stable across the independent runs, as other methods have significantly larger deviation. Finally, Figure 4 depicts the confusion matrix, PR and ROC curves, as well as objective indicator joint plot of the suggested HASCA algorithm.

### 5.2 Experiment 2 - Multiclass classification

The simulation outcomes of the proposed RNN-HASCA method and seven competitor methods for the mutliclass classification problem are summarized in Table 4. Similarly to the first experiment, it is worth highlighting that the objective function was classification error, and the scores of the Cohen’s Kappa coefficient are provided as well. When observing the overall metrics of the objective function across 30 independent executions, shown in Table 4, it is possible to note that the proposed HASCA algorithm exhibited superior performance, achieving the best scores for best, worst and mean metrics, as well as for standard deviation and variance. SCA attained the second best score, and COLSHADE finished third. Regarding the worst metric, SCA again finished second, behind HASCA. Additionally, regarding the mean metrics, SCA attained second place, while COLSHADE finished third. It is also worth noting that RNN-HASCA method obtained the best scores for Cohen’s Kappa coefficient as well.

**TABLE 4:** Experiment 2 - overall objective and Cohen Kappa metrics for 30 runs.

Method	Best	Worst	Mean	Median	Std	Var
RNN-HASCA	**0.034370**	**0.124386**	**0.054874**	0.124386	**2.64E-02**	**6.99E-04**
RNN-SCA	0.047872	0.139525	0.103928	0.124386	4.01E-02	1.61E-03
RNN-GA	0.146481	0.243453	0.204719	0.224223	4.19E-02	1.76E-03
RNN-PSO	0.101064	0.321604	0.222995	0.246318	9.15E-02	8.38E-03
RNN-FA	0.224223	0.386661	0.300600	0.290917	6.67E-02	4.44E-03
RNN-BSO	0.224223	0.332242	0.271686	0.258592	4.51E-02	2.03E-03
RNN-RSA	0.132160	0.319558	0.219585	0.207038	7.70E-02	5.93E-03
RNN-COLSHADE	0.087152	0.224223	0.136661	**0.098609**	6.21E-02	3.86E-03
Cohen Kappa metrics						
RNN-HASCA	**0.910288**	**0.708847**	**0.863479**	**0.878882**	5.89E-02	3.47E-03
RNN-SCA	0.878882	0.652979	0.746903	0.708847	9.61E-02	9.23E-03
RNN-GA	0.643423	0.208901	0.284108	0.208901	2.68E-01	7.18E-02
RNN-PSO	0.755973	0.167649	0.297728	0.167649	3.34E-01	1.12E-01
RNN-FA	0.245910	−0.028157	0.072584	0.000000	1.23E-01	1.52E-02
RNN-BSO	0.000000	−0.103654	−0.043166	−0.025843	**4.41E-02**	**1.94E-03**
RNN-RSA	0.710155	0.411494	0.502563	0.411494	1.47E-01	2.17E-02
RNN-COLSHADE	0.788920	0.000000	0.516435	0.760384	3.65E-01	1.33E-01

In all tables that contain simulation results, the best score in every category is marked bold.

Detailed metrics achieved in the best individual run of every observed method are provided in Table 5. It must be highlighted that in this scenario (multiclass classification), suggested HASCA obtained superior results, with achieved accuracy of 96.56%. Finally, Table 6 depicts the best set of RNN hyperparameters obtained by each algorithm. It is noteworthy that HASCA again determined the network with one layer.

**TABLE 5:** Experiment 2 - detailed metrics of the best run of each algorithm.

Metric	RNN-HASCA	RNN-SCA	RNN-GA	RNN-PSO	RNN-FA	RNN-BSO	RNN-RSA	RNN-COLSHADE
Accuracy (%)	**96.563**	31.3830	85.3519	89.8936	77.5777	77.5777	86.7840	91.2848
Precision Normal	**0.902148**	0.158545	0.521622	0.862155	0.000000	0.000000	0.874439	0.856492
Precision Anomaly	**0.830601**	0.069825	0.000000	0.420195	0.000000	0.000000	0.348780	0.492537
Precision Seizure	0.993485	0.779940	0.997653	0.991945	0.775777	0.775777	**1.000000**	0.991940
W.Avg. Precision	**0.968380**	0.634874	0.857194	0.934271	0.601831	0.601831	0.937864	0.938041
Recall Normal	0.969231	0.558974	0.989744	0.882051	0.000000	0.000000	**1.000000**	0.964103
Recall Anomaly	**0.962025**	0.177215	0.000000	0.816456	0.000000	0.000000	0.905063	0.835443
Recall Seizure	0.965190	0.274789	0.896624	0.909283	1.000000	1.000000	0.837553	0.908755
W.Avg. Recall	**0.965630**	0.31383	0.853519	0.898936	0.775777	0.775777	0.867840	0.912848
F1-score Normal	0.969231	0.558974	0.989744	0.882051	0.000000	0.000000	**1.000000**	0.964103
F1-score Anomaly	**0.962025**	0.177215	0.000000	0.816456	0.000000	0.000000	0.905063	0.835443
F1-score Seizure	0.965190	0.274789	0.896624	0.909283	1.000000	1.000000	0.837553	0.908755
W.Avg. F1-score	**0.965630**	0.31383	0.853519	0.898936	0.775777	0.775777	0.867840	0.912848

In all tables that contain simulation results, the best score in every category is marked bold.

**TABLE 6:** Experiment 2 - best determined RNN parameters.

Method	Learning rate	Dropout	Epochs	Number of layers	Layer 1	Layer 2
RNN-HASCA	0.001781	0.200000	150	1	30	30
RNN-SCA	0.003387	0.142190	139	2	22	22
RNN-GA	0.001236	0.088814	50	2	30	9
RNN-PSO	0.001725	0.115795	50	2	18	11
RNN-FA	0.000100	0.200000	138	1	23	16
RNN-BSO	0.000100	0.200000	120	2	21	13
RNN-RSA	0.000945	0.105523	150	2	30	13
RNN-COLSHADE	0.001196	0.200000	101	2	18	30

**FIGURE 5:**
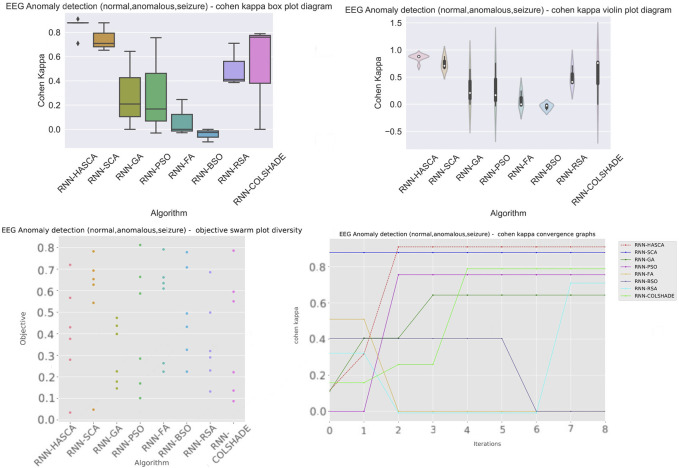
Experiment 2 - box plot, violin plot, swarm diversity diagram and objective convergence diagram.

**FIGURE 6:**
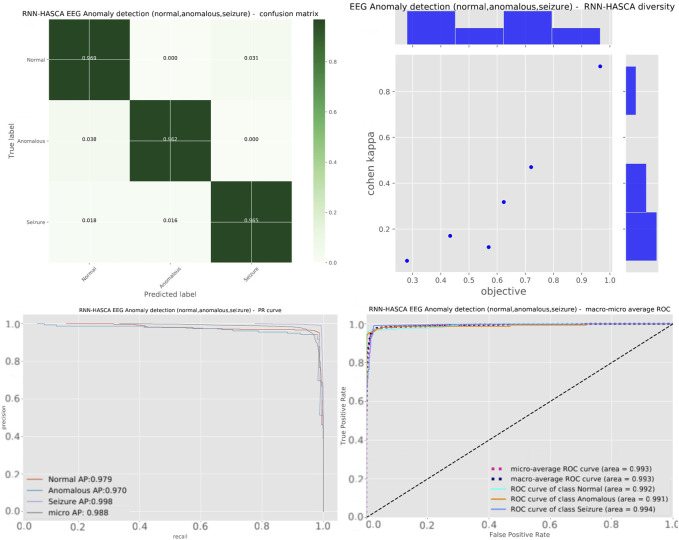
Experiment 2 - confusion matrix, objective indicator joint plot, PR and ROC curves of the proposed HASCA method.

Aiming to present the obtained results clearer, Figure 5 shows the box plots, violin plots, convergence diagram and swarm diversity plots. It can be observed that the HASCA method exhibits excellent converging speed, and also the box plots show that the results are very stable across the independent runs, as other methods have significantly larger deviation. Finally, Figure 6 depicts the confusion matrix, PR and ROC curves, as well as objective indicator joint plot of the suggested HASCA algorithm.

### 5.3 Statistical validation

To facilitate statistical analysis, the best samples were captured from 30 independent runs of each optimizer. Following this step, the safe use of parametric tests needed to be justified. To accomplish this, several criteria needed to be fulfilled (LaTorre et al., 2021) including independence, normality, and homoscedasticity of the data variance.

The initial independence condition is fulfilled, as each algorithm is initialized with a different random seed and accordingly a new set of random solutions is generated. To determine if the normality condition is met, the Shapiro-Wilk (Shapiro and Francia, 1972) test for individual problem analysis is used for both binary and multi-class experiments’ objective function outcomes. Testing is independently conducted for each algorithm and the determined *p*-values can be observed in Table 7.

**TABLE 7:** Shapiro Wilk normality tests.

Problem	HASCA	SCA	GA	PSO	FA	BSO	RSA	COLSHADE
Experiment 1	0.046	0.047	0.044	0.041	0.048	0.045	0.044	0.042
Experiment 2	0.029	0.035	0.030	0.027	0.031	0.033	0.036	0.029

Based on the null hypothesis (H0) that the data originates form a normal distribution, the *p*-values attained by the Shapiro Wilk shown in Table 7 indicate that the samples for Experiment 1 originate for a near-normal distribution, while samples for Experiment 2 deviate significantly. Nevertheless, as non of the samples meet the normality condition with all *p*-values below the 0.05 H0 can be rejected.

As the outcomes of the Shapiro-Wilk normality tests indicate that the samples do not fulfill the normality condition, the safe use of parametric tests is not justified. Therefore, the non-parametric Wilcoxon signed-rank test (Wilcoxon, 1992) is utilized. For this analysis, the proposed RNN-HASCA approach is utilized as the control. The outcomes of the Wilcoxon signed-rank test are shown in Table 8.

**TABLE 8:** Wilcoxon signed-rank test scores representing *p*-values for all four scenarios (XG-MFA vs. others).

Problem/*p*-values	SCA	GA	PSO	FA	BSO	RSA	COLSHADE
Experiment 1	0.036	0.036	**0.053**	0.033	0.033	0.037	0.028
Experiment 2	0.038	0.029	0.026	0.016	0.021	0.027	0.034

In all tables that contain simulation results, the best score in every category is marked bold.

The outcomes provided in Table 8 indicate that statistical significance criteria are met in most cases except in one. In experiment 1, the introduced metaheuristic does not show a statistically significant improvement when compared to the PSO algorithm. This can be due to a positioning advantage attained by the PSO algorithm during randomized initialization. An additional observation can be made concerning experiment one, where several algorithms attained matching *p*-values, which can be further confirmed by observing preceding results indicating that these algorithms attain similar objective function outcomes during experimenting. Nevertheless, in all cases except the PSO in experiment 1, the improvements of the introduced algorithm are noticeable and statistically significant.

### 5.4 Best model interpretation

Traditionally, AI algorithms have often been treated as a black box. While with simple models further importance can be determined empirically, as model complexity increases, interpretability becomes more difficult. In recent years more focus has been placed on interpreting model decisions. Interpretation techniques help researchers understand and debug models but also provide a deeper understanding of the influence of features on model decisions and therefore outcomes. One promising technique that leverages model approximations to determine feature importances is SHapley Additive exPlanations (SHAP) (Lundberg and Lee, 2017). This approach relies on concepts from game theory to interpret and explain the output of any ML model.

**FIGURE 7:**
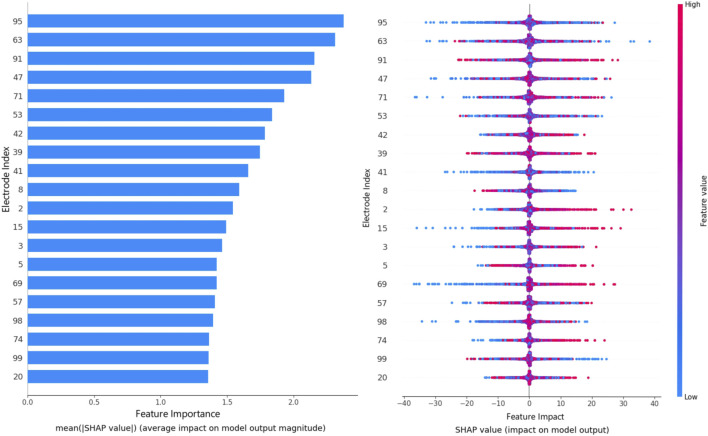
SHAP interpretation outcomes for feature impacts.

In this work, SHAP interpretation has been leveraged to interpret the best-performing classification models generated by metaheuristics. These interpretations can prove useful for future research suggesting what segments of the available data can be improved or improved to augment future research. Additionally, the interpretations can prove critical for diagnostic works, reducing the number of electrodes needed to determine outcomes. The interpretation of the best-performing model optimized by metaheuristics is provided in Figure 7.

Shown in Figure 7 are the importance each electrode plays in the classification of the best prediction model, as well as the ranges in which these features impact a decision towards a normal or abnormal outcome. As it can be observed the impacts of all features is reasonable. However, electrodes labeled 95, 63, 91, and 47 play a more significant role in the decision-making process of the model.

## 6 Conclusion

The research provided in this manuscript focused on medical EEG dataset classification, through application of the hybrid machine learning and metaheuristics approach. First, a novel hybrid variant of the well-known SCA metaheuristics was introduced, where the deficiencies of the baseline SCA were addressed by adding a chaotic initialization of the population, and incorporating FA search to enhance the exploration. The devised metaheuristics was named HASCA, and it was later employed to tune the hyperparameters of the RNN.

The RNN-HASCA method was evaluated on EEG medical dataset, and the results have been compared to the performance of other contending cutting-edge metaheuristics algorithms. The introduced algorithm attained an accuracy of 99.8769%, The simulation outcomes unambiguously indicate the superiority of the suggested method, that was also validated by applying thorough statistical analysis of the simulation results. The statistical tests have shown that the RNN-HASCA performs statistically significantly better than other regarded methods. Finally, the top-performing model was subjected to SHAP analysis, in order to interpret the results, and better understand the influence of the features on model decisions. The outcome of the SHAP analysis can be leveraged to reduced the number of features needed for detection and thus reduce the computational demands of constructed classification models.

Future work in this domain should focus in two directions. First, the applications of the proposed methodology should be examined further by testing it on other medical data structured as time series, such as electrocardiogram (ECG). The second direction would include examining the proposed HASCA method’s capabilities in tuning the hyperparameters of other machine learning models in other application domains, such as intrusion detection, image classification and stock predictions.

## Data Availability

The original contributions presented in the study are included in the article/Supplementary Materials, further inquiries can be directed to the corresponding author.

## Footnotes

1
https://www.upf.edu/web/ntsa/downloads

2
https://www.upf.edu/web/ntsa/downloads

## Supplementary material

Supplementary Data Sheet S1: EEG data with 2 classes (**A** and **C**)- binary classification.

Supplementary Data Sheet S2: EEG data with 3 classes (**A**, **D** and **E**)-multiclass classification.

## Abbreviations

EEG, electroencephalography; AI, artificial intelligence; RNN, recurrent neural networks; ANN, artificial neural networks; SCA, sine cosine algorithm; HASCA, hybrid adaptive sine cosine algorithm; SHAP, SHapley Additive exPLanations.
